# Obstetric violence and its associated factors among postnatal women in a Specialized Comprehensive Hospital, Amhara Region, Northwest Ethiopia

**DOI:** 10.1186/s13104-019-4614-4

**Published:** 2019-09-18

**Authors:** Muhabaw Shumye Mihret

**Affiliations:** Department of Clinical Midwifery, School of Midwifery, College of Medicine and Health Sciences, University of Gondar, Po. Box 196, Gondar, Ethiopia

**Keywords:** Ethiopia, Gondar university hospital, Obstetric violence, Respectful maternity care

## Abstract

**Objective:**

Obstetric violence is an often overlooked obstacle to quality maternal health care service utilization. In the study setting, there was limited evidence on obstetric violence. Hence, this study aimed at assessing the prevalence and associated factors of obstetric violence among women who gave birth in Gondar University Specialized Comprehensive Hospital, Northwest Ethiopia.

**Results:**

A total of 409 women had been participated in the study with a response rate of 100%. Three in four (75.1%) women reported that they had been subjected to at least one form of obstetric violence during labor and delivery with 95% CI (70.9–79.0). The reported forms of obstetric violence include non-consented care—260 (63.6%), non-dignified care—226 (55.3%), physical abuse—192 (46.9%), non-confidential care—132 (32.3%, neglected care—52 (12.7%) and discriminated care—38 (9.3%). On the contrary, none of the respondent had reported detention for failure to pay in the hospital. The multivariable logistic regression analysis demonstrated that urban residents (AOR = 1.89; 95% CI 1.11, 3.22) and primary school attendants (AOR = 0.49; 95% CI 0.27, 0.91) were significantly associated with experiencing obstetric violence. This study indicated the high prevalence of obstetric violence. Thus, interventions need to be undertaken by taking the reported forms of obstetric violence and participants’ sociodemographic status in to account.

## Introduction

Obstetric violence (OV) is a specific type of violation of women’s rights in medical practice during health care related to the child birth processes [1]. Laboring mothers may be subjected to different forms of OV during facility child birth [2]. Such ill-treatments and abuses create a psychological distance between the women and care providers and then drive women away from formal health care systems in fear of being subjected to such violence and sometimes are a more prominent hindrance than geographical or financial barriers to maternal health service utilization [1, 3–5]. Despite this fact, OV is also an often overlooked type of obstacle to quality maternal health care service utilization as compared to other barriers [1, 6].

Maternal mortality (MM) remains high in many resource limited countries including Ethiopia [6–8]. To reduce such high maternal mortality (MM) and thereby to achieve target two of Sustainable Development Goal (SDG) three [9], one key identified strategy is improving access to facility delivery [10–13]. Unfortunately, only one out of four women have undergone institutional delivery in Ethiopia [4, 7]. In this perspective, an important but little understood concept that retards the universal access to facility delivery is OV [2, 14, 15]. Thus, it is essential to conduct investigation on OV and its determinants. Previous studies demonstrated that OV had been predicted by factors like educational status, long stay in the hospital and residency [14–17]. However, these determinants could be varied across settings and time trend.

On top of this, there was limited evidence on OV in the study setting. Therefore; this study aimed at assessing the prevalence and associated factors of OV among immediate postnatal women in care in Gondar University Comprehensive Specialized Hospital (GUCSH), Northwest Ethiopia, 2019.

## Main text

### Methods and materials

An institutional based cross sectional study was conducted from April 08 to May 16/2019 in GUCSH which is located 750 km from the northwest of Addis Ababa—the capital city of Ethiopia. The hospital is providing service for more than 7 million people of Gondar city and the catchment areas. The maternity division of the hospital is composed of antenatal care, postnatal care and three maternity wards (named as M1, M2 and M3). It is also staffed with intern doctors, Midwives, Obstetricians and supportive staffs. In 2018, a total of 9744 births were attended at the hospital. Women who gave birth at GUCSH and got discharge decision (i.e. to prevent under report of OV in their entire hospital stay) during the study period were included. Mothers who had ever experienced loss of consciousness during the hospital stay were excluded to minimize recall bias.

Sample size was initially determined for both specific objectives using Open-Epi Version 2 software (Additional file 1: Annex S1). Thereafter, the largest sample size had been taken. Thus, the current study’s final sample size had been made by considering the following assumptions: ratio between women with monthly family income < 2000 and ≥ 2000 Ethiopian Birr—1:1, odds ratio—1.90, power—80%, confidence interval—95%, and the proportion of OV among exposed group—73.5% and that of among non-exposed group—59.2% [15]. Therefore, the largest sample size was 368 and by assuming 10% non-response rate, the final sample size (i.e. 368 * (1/1 ‒ 0.10)) turned to be 409.

Systematic random sampling was employed based on respondents’ discharge decision order. The K-interval was made by dividing the number of cases flow in the preceding month (which was 863) by the sample sizes (i.e. 860/409 ~ 2). Lottery method was then used to pick the first participant among the first two women who had been earned decision for discharge. Thereafter, selection of participants had been proceeded every two interval.

The outcome variable was obstetric violence (OV) while the explanatory variables included socio-demographic and obstetric variables.

Obstetric violence (OV) was measured using the seven performance standards and their respective verification criteria developed by the Maternal and Child Health Integrated Program [18]. The seven categories of OV include physical abuse, non-consented care, non-confidential care, non-dignified care, discriminated care, neglected care and detention in the health facility. Accordingly, those women who replied ‘yes’ to at least one form of OV had been labeled to be subjected to OV. A total of 25 verification criteria of OV were used to measure the seven performance standards in the composite scale (Additional file 2: Annex S2).

Structured pretested questionnaire through face to face interview was used to collect the data. The questionnaire was designed in English and translated in to Amharic (local) language and then translated back to English to check for consistency. One day training was provided for the data collectors and supervisors. On daily basis, data were checked for completeness and accuracy and then necessary feedback was offered. All the questionnaires were checked for completeness, coded and entered in to Epi Info version 7.1.2.0 and then exported to SPSS version 20 for analysis. Descriptive statistics such as mean, percentage and standard deviation were employed. Both bivariate and multivariable logistic regression model were fitted. COR and AOR with 95% CI were computed. Finally, statistically significant association of variables was claimed based on AOR with its 95% CI and p-value ≤ 0.05.

### Results

#### Socio-demographic characteristics

A total of 409 immediate postnatal women were interviewed making a response rate of 100%. Majority (84.8%) of the respondents’ age ranged 20–34 years with mean ± SD of 28.1 ± 5.02 years. About 394 (96.3%) of mothers were married and more than half (56.2%) of the participants were housewife by occupation. About 131 (32.03%) of women had never attended any formal education whereas nearly one-third (34.7%) of women had attended college and above (Table 1).

**Table 1:** Socio-demographic characteristics of participants in Gondar University Comprehensive Specialized Hospital, Northwest Ethiopia, 2019 (n = 409)

Variables	Number	%
Age		
18–19	10	2.5
20–34	347	84.8
≥ 35	52	12.7
Marital status		
Married	396	96.82
Divorced	7	1.71
Single	5	1.22
Widowed	1	0.25
Religion		
Orthodox	301	73.6
Muslim	90	22.0
Protestant	18	4.4
Ethnicity		
Amhara	356	87.0
Qimannt	31	7.6
Tigray	16	3.9
Oromo	6	1.5
Educational attendance		
Unable read and write	63	15.4
Can read and write	68	16.6
Primary school (grade 1–8)	69	16.9
Secondary school (grade 9–12)	67	16.7
Collage and above	142	34.7
Occupation		
House wife	230	56.2
Government employee	118	28.9
Private business	52	12.7
Other^a^	9	2.2
Husband’s educational status (n = 396)		
Unable read and write	39	9.5
Can read and write	40	9.8
Primary school (grade 1–8)	52	12.7
Secondary school (grade 9–12)	66	16.1
College and above	199	48.7
Husband’s occupation (n = 396)		
Farmer	61	15.4
Private business	126	31.82
Government employee	165	41.67
Daily laborer	44	11.11
Families’ monthly income (ETB)		
≤ 2500	62	15.2
> 2500	347	84.8
Residence		
Urban	282	68.9
Rural	127	31.1

^a^Others = student and daily laborer

#### Obstetric characteristics

Majority (97.1%) of respondents had received ANC service during their most recent pregnancy. Of them: nearly two-third (66.5%) of the mothers received ANC by Midwives and almost two-third (65.5%) of the participants attended complete ANC visits (Table 2).

**Table 2:** Obstetric characteristics of participants in Gondar University Comprehensive Specialized Hospital, Northwest Ethiopia, 2019 (n = 409)

Variable	Number	%
ANC		
Yes	397	97.1
No	12	2.9
Place of ANC (n = 397)		
Health center	199	48.7
Hospital	181	44.3
Private health facility	17	4.2
ANC received from (n = 397)		
Doctors	76	18.6
Midwives	272	66.5
Inter doctors	48	11.7
Other	1	0.2
Number of ANC visit (n = 397)		
≥ 4	260	65.5
< 4	137	34.5
Gravidity		
1	115	28.1
2–3	211	51.6
≥ 4	83	20.3
Parity		
Prim parous	128	31.3
Multi parous	253	61.9
Grand multiparous	28	6.8
History of previous institutional delivery		
Yes	219	53.5
No	190	46.5
Number of birth attendants		
1–2	275	67.2
3–4	116	28.4
5–8	18	4.4
Main birth attendant		
Midwife	193	47.2
Doctor	157	38.4
Intern doctor	59	14.4
Sex of main birth attendant		
Female	158	38.6
Male	251	61.4
Rout/type of current delivery		
Spontaneous vaginal delivery (SVD)	242	59.2
Assisted vaginal delivery (AVD)	35	8.6
Delivery by episiotomy	77	18.8
Caesarean delivery (CD)	55	13.4
Preferred birth position		
Kneeling	212	51.8
Squatting	43	10.5
Lithotomic	154	37.7
Labor was augmented/induced		
Yes	155	37.9
No	254	62.1
Transfused with blood		
Yes	19	4.6
No	390	95.4
Time of delivery		
Day	270	66
Night	139	34
Number of days stayed at the hospital after delivery		
≤ 3	328	80.2
> 3	81	19.8

#### Prevalence and forms of obstetric violence

In the study setting, the overall prevalence of OV found to be 75.1% with 95% CI (70.9–79.0). Six out of seven forms of OV had been reported by the study participants. The most common one was non-consented care—260 (63.6%) (Additional file 3: Figure S1).

#### Correlates of obstetric violence

Initially, all explanatory variables had been fitted to bivariate logistic regression model. Accordingly; residence and educational status had been associated with OV. Thereafter, variables with missing values had been thrown out from the multivariable regression. In the multivariable analysis, residence and educational status had been still remained significantly correlated with OV.

Thus, urban residents were 1.89 times more likely to report OV as compared to rural dwellers (AOR = 1.89; 95% CI 1.11, 3.22) whereas women who had attended primary school were 51% less likely to report obstetric violence as compared to those women who had attended secondary education and above (AOR = 0.49; 95% CI 0.27, 0.91) (Table 3).

**Table 3:** Correlates of obstetric violence among mothers in care in Gondar University Comprehensive Specialized Hospital, Northwest Ethiopia, 2019 (n = 409)

Variable	Obstetric violence		COR with 95% CI	AOR with 95% CI
	Yes N (%)	No N (%)		
Age				
≤ 34	264 (73.9)	93 (26.1)	0.59 (0.28, 1.27)	0.49 (0.22, 1.11)
> 34	43 (82.7%)	9 (17.3)	1	1
Residence				
Urban	220 (78)	62 (22)	1.63 (1.02, 2.61)	1.89 (1.11, 3.22)
Rural	87 (68.5)	40 (31.5)	1	1
Religion				
Christian	238 (74.6)	81 (25.4)	0.89 (0.52,1.55)	
Muslim	69 (76.7)	21 (23.3)	1	
Educational status				
No formal education	101 (77.1)	30 (22.9)	0.98 (0.58, 1.65)	1.18 (0.65, 2.13)
Primary school	44 (63.8)	25 (36.2)	0.51 (0.28, 0.92)	0.49 (0.27, 0.91)*
Secondary school (+)	162 (77.5)	47 (22.5)	1	1
Occupation				
Housewife	172 (74.8)	58 (25.2)	0.92 (0.55, 1.55)	
Private business	45 (73.8)	16 (26.2)	0.88 (0.43, 1.78)	
Governmental employed	90 (76.3)	28 (23.7)	1	
Monthly income				
≤ 2500	43 (69.4)	19 (30.6)	0.71 (0.39, 1.29)	
> 2500	264 (76.1)	83 (23.9)	1	
Number of birth attendants				
1–2	208 (75.6)	67 (24.4)	1.10 (0.68, 1.76)	
≥ 3	99 (73.9)	35 (26.1)	1	
Parity				
Primiparous	93 (72.7)	35 (27.3)	0.83 (0.52, 1.34)	
Multiparous	214 (76.2)	67 (23.8)	1	
Mode of delivery				
SVD	185 (76.4)	57 (23.6)	1.20 (0.76, 1.88)	
C/S	122 (73.1)	45 (26.9)	1	
Staying hour				
≤ 24	223 (74.1)	78 (25.9)	0.82 (0.49, 1.38)	
> 24	84 (77.8)	24 (22.2)	1	
History of previous facility delivery				
No	137 (72.1)	53 (27.9)	0.75 (0.48, 1.17)	0.71 (0.44, 1.14)
Yes	170 (77.6)	49 (22.4)	1	1
Person attending labor				
Midwives	141 (73.1)	52 (26.9)	0.77 (0.38,1.53)	
Obstetricians (resident+)	120 (76.4)	37 (23.6)	0.92 (0.45, 1.88)	
Interns	46 (78)	13 (22)	1	
Time of birth				
Day	199 (73.7)	71 (26.3)	0.81 (0.50, 1.30)	
Night	108 (77.8)	31 (22.3)	1	
Birth attendant’s gender				
Female	117 (74.1)	41 (25.9)	0.92 (0.58, 1.45)	
Male	190 (75.7)	61 (24.3)	1	

### Discussion

This study aimed to assess obstetric violence and associated factors among women during childbirth in the study setting. The study exhibited high prevalence of OV and was significantly associated with urban resident and primary school attendant. In this study, three out of four (75.1%) women reported to be subjected to at least one form of OV. The prevalence of OV in the current study is consistent with that of the previous studies done in Ethiopia such as Addis Ababa—78% [19] and Western Ethiopia—74.8% [20].

However, the proportion of OV in this study was higher than other previous studies’ report conducted in north Nigeria—55.9% [17], Kenya—20% [21], Brazil—18.3% [22], Northern India—28.8% [23] and Uttar Pradesh India—15.2% [24]. It was also higher as compared to studies done in Ethiopia such as pooled prevalence of systematic review and meta analysis—49.4% [25], Bahirdar city—67.1% [15], Southern Ethiopia—21.1% [26], four regions’ observational report—36% [27] and Northern Ethiopia—22% [16]. This discrepancy might be due to variation in sample sizes [16, 17, 22, 26–28]. Furthermore, the disagreement could be ascribed to difference in study settings. Most of the aforementioned studies were conducted in low level settings with low clients flow unlike the current tertiary level setting with higher cases flow and abundant referral complicated cases. With overcrowded and complicated cases, care providers are likely to behave abusively. Equally, variation in the study period might contribute for such inconsistent magnitude [21, 26, 27, 29]. In addition, variation in time of interview after child birth could be another reason for the observed difference in prevalence [22].

On the contrary, the prevalence of OV in the present study is lower than was reported in previous study conducted in Jimma University medical center in southwest Ethiopia—91.7% [14]. This disparity could be due to study population variation that the former study had excluded women who gave birth via caesarean section. In this perspective, the empirical evidence support that more OV had been reported among women who gave birth through vagina than caesarean section [30]. This could be due to the fact that health care providers usually tend to pay more attention for women who are indicated for caesarean delivery (for example, more likely to take informed consent strictly) than vaginal delivery thereby minimized committing obstetric OV. The current study’s report also lower than that of reported in the study undertaken in Malawi—93.7% [28]. A number of reasons could be mentioned for the observed inconsistency. One reason might be related to variation in data collection method. The data in the current study were collected through interviewing the study participants while data were collected via direct observation in the Malawi’s study. Hence, the magnitude of obstetric OV when it collected through interview might be affected by participants’ recalls capacity and level of perception. Another possible reason would be related to time of data collection since the previous data were collected 5 years back and thus, provider’s awareness and quality of care tends to be improved across time trend. In addition, variation in sample sizes, study settings and verification criteria for the outcome variable might attribute for the observed disagreement.

The analysis in the current study revealed that women who have been living in the urban were 1.89 times more likely to report at least one form of OV than the rural dwellers. This finding is in line with the study done in Tigray in Northern Ethiopia [16]. It could be explained by the women’s level of awareness on their rights that women who have been living in rural area are less likely to be aware. In this regard, evidence shows that women who are not aware of their rights and have never been exposed to any other system of care are not sensitive to the disrespect and abuse of health care workers and see such behavior and attitude as status [5]. Hence, rural resident women are not likely to report being subjected to OV.

This study also shows that there is a significant association between educational status of the respondents and the OV. Accordingly, women who had attended only primary education were 51% less likely to report any form of OV than those who had attended secondary education and above. This finding is in accordance with previous studies done in Jimma University Medical Center in Southwest Ethiopia [14] and Tigray in Northern Ethiopia [16]. This independent association could convince since educated women are better aware of their rights and thus are more perceptive enough to report all likelihood of being subjected to any form of OV.

### Conclusion

This study demonstrated high prevalence of OV in the study setting and was significantly associated with urban residents and primary school attendants. The top three common reported forms of OV were non-consented care, non-dignified care and physical abuse. Thus, interventions need to be undertaken by taking the reported forms of obstetric violence and participants’ sociodemographic status in to account.

## Limitations

This study was entirely made based on interview rather than observation. In addition, the author recommended further studies to be carried out using a qualitative approach.

## Supplementary information

**Additional file 1: Annex S1.** Sample size determination.

**Additional file 2: Annex S2.** Verification criteria for the seven performance standards (categories of obstetric violence) which employed to measure obstetric violence [16].

**Additional file 3: Figure S1.** Forms of obstetric violence reported by participants in Gondar University Comprehensive and Specialized Hospital, Northwest, Ethiopia, 2019.

## Data Availability

The datasets employed in the current study can be available from the corresponding author upon the reasonable request.

## Abbreviations

GUCSH: Gondar University Comprehensive Specialized Hospital
OV: obstetric violence
