# Supplementary material for: Obstetric violence and its associated factors among postnatal women in a Specialized Comprehensive Hospital, Amhara Region, Northwest Ethiopia
Source: BMC Res Notes. 2019 Sep 18;12:600. doi: 10.1186/s13104-019-4614-4 (PMC6751597; doi:10.1186/s13104-019-4614-4)
Supplement: Supplementary file 1 — Additional file 1: Annex S1. Sample size determination. [file 13104_2019_4614_MOESM1_ESM.docx]

## Annex S1: Sample size determination

**Sample size determination for proportion (For the first objective)**

Sample size for the study was determined using an Open-Epi Version 2, software by considering the following assumptions; 67.1% the proportion of mothers who experienced obstetric violence during child birth (1), 95% level of confidence, and 5 % margin of error. Thus, the sample size of 340 was obtained.

**Sample size determination for associated factors** **(For the second Objective)**

The table below summarizes the sample size determination for factors associated with obstetric violence among mothers who gave birth at Gondar university specialized comprehensive hospital, Amhara region, Northwest Ethiopia, 2019 (1).

| S/No | Factors | Assumptions | | | | | | | |
| --- | --- | --- | --- | --- | --- | --- | --- | --- | --- |
|  |  | Ratio | Power % | CI % | COR | Proportion of obstetric violence among exposed | Proportion of obstetric violence among non-exposed | Design  Effect | Sample size |
| 1 | Number of ANC visit | 1:1 | 80 | 95 | 2.43 | 79.4 | 61.3 | 1 | 220 |
| 2 | Long stay at health facility | 1:1 | 80 | 95 | 7.10 | 89.9 | 55.5 | 1 | 62 |
| 3 | Monthly family income | 1:1 | 80% | 95 | 1.90 | 73.5 | 59.2 | 1 | 368 |

So, the largest sample size for this study is 368 and by assuming 10% non-response rate, the final sample size =368*(1/1-0.10) =409.
