# Supplementary material for: Obstetric violence and its associated factors among postnatal women in a Specialized Comprehensive Hospital, Amhara Region, Northwest Ethiopia
Source: BMC Res Notes. 2019 Sep 18;12:600. doi: 10.1186/s13104-019-4614-4 (PMC6751597; doi:10.1186/s13104-019-4614-4)
Supplement: Supplementary file 2 — Additional file 2: Annex S2. Verification criteria for the seven performance standards (categories of obstetric violence) which employed to measure obstetric violence [16]. [file 13104_2019_4614_MOESM2_ESM.docx]

**Annex 2: Verification criteria for the seven performance standards (categories of obstetric violence) which employed to measure obstetric violence [16].**

**Physical abuse**: The presence of at least one of the following activities by the care provider on the client: beating, threatening with beating, slapping, pinching, restraining or tying down during labor, cutting or suturing of episiotomy cuts or perineal tears without the use of anesthesia and the use of fundal pressure to fasten the delivery of the baby. A woman who answered ‘yes’ to at least one of the criteria was considered as physically being abused at the time of labor and delivery.

**Non-consented care:** The presence of at least one of the following: providers not giving women or her relatives proper information about medical procedures, not asking for women’s permission to conduct medical procedures such as cesarean sections, episiotomies, hysterectomies, blood transfusions, tubal ligation, augmentation of labor; and coercing into a medical procedures such as a cesarean section. A woman who answered ‘ yes’ to at least one of the criteria was considered as being abused in non-consented care at the time of labor and delivery.

**Non-confidential care**: The presence of at least one of the following: giving birth in a public view without privacy barriers such as curtains; and having healthcare providers share sensitive clients’ information, such as HIV status, age, marital status, and medical history, in a way that other people who are not involved in their care can hear. A woman who replied ’ yes’ to at least one of the criteria was considered as being abused in non-confidential care at the time of labor and delivery.

**Non-dignified care:** A report by the client about at least one of the following: intentional humiliation, blaming, rough treatment, scolding, shouting at, women not allowed to bring companion to the labor ward, and ordering to stop crying while they are in labor pain. A woman who replied ‘yes’ to at least one of the criteria was considered as being abused in non-dignified care at the time of labor and delivery.

**Discrimination:** Discrimination based on specific client attributes like race, age, HIV/AIDS status, traditional beliefs and preferences, economic status, or educational background. A woman who answered yes to at least one of the criteria was considered as being abused in discriminated care at the time of labor and delivery.

**Neglected care**: If there was any of the following practices: leaving laboring woman alone, women giving birth by themselves at health facilities, failure of care givers to monitor women in labor and intervene in life threatening conditions. A women who answered ‘yes’ to at least one of the criteria was considered as being abused in neglected care at the time of labor and delivery.

**Detention in facilities:**  detaining of mothers in health facility because of bills or damage to the property of the health care facility. If a woman answered ‘yes’ to at least two of the criteria, then she would be considered as being abused at the time of labor and delivery.
