# Supplementary material for: Obstetric violence and its associated factors among postnatal women in a Specialized Comprehensive Hospital, Amhara Region, Northwest Ethiopia
Source: BMC Res Notes. 2019 Sep 18;12:600. doi: 10.1186/s13104-019-4614-4 (PMC6751597; doi:10.1186/s13104-019-4614-4)
Supplement: Supplementary file 3 — Additional file 3: Figure S1. Forms of obstetric violence reported by participants in Gondar University Comprehensive and Specialized Hospital, Northwest, Ethiopia, 2019. [file 13104_2019_4614_MOESM3_ESM.docx]

Fig S1
